# Analysis of Alternative Shelf Life-Extending Protocols and Their Effect on the Preservation of Seafood Products

**DOI:** 10.3390/foods11081100

**Published:** 2022-04-12

**Authors:** Lourenço Pinto de Rezende, Joana Barbosa, Paula Teixeira

**Affiliations:** Laboratório Associado, Escola Superior de Biotecnologia, CBQF—Centro de Biotecnologia e Química Fina, Universidade Católica Portuguesa, 4169-005 Porto, Portugal; pinto.rezende88@gmail.com (L.P.d.R.); pcteixeira@ucp.pt (P.T.)

**Keywords:** biopreservation, edible coatings, high-pressure, hyperbaric storage, superchilling

## Abstract

Seafood is essential to a healthy and varied diet due to its highly nutritious characteristics. However, seafood products are highly perishable, which results in financial losses and quality concerns for consumers and the industry. Due to changes in consumer concerns, demand for healthy products has increased. New trends focusing on reducing synthetic preservatives require innovation and the application of additional or alternative strategies to extend the shelf life of this type of product. Currently, refrigeration and freezing storage are the most common methods for fish preservation. However, refrigeration alone cannot provide long shelf-life periods for fish, and freezing worsens sensorial characteristics and consumer interest. Therefore, the need to preserve seafood for long periods without exposing it to freezing temperatures exists. This review focuses on the application of other approaches to seafood products, such as biodegradable films and coating technology; superchilling; irradiation; high-pressure processing; hyperbaric storage; and biopreservation with lactic acid bacteria, bacteriocins, or bacteriophages. The efficiency of these techniques is discussed based on their impact on microbiological quality, sensorial degradation, and overall preservation of the product’s nutritional properties. Although these techniques are already known, their use in the industrial processing of seafood is not widespread. Thus, the novelty of this review is the aggregation of recent studies on shelf life extension approaches, which provide useful information for the selection of the most appropriate technology and procedures and industrial innovation. Despite the fact that all techniques inhibit or delay bacterial proliferation and product decay, an undesirable sensory impact may occur depending on the treatment conditions. Although no technique appears to replace refrigeration, the implementation of additional treatments in the seafood processing operation could reduce the need for freezing, extending the shelf life of fresh unfrozen products.

## 1. Introduction

The global seafood market has witnessed a steady growth lately, representing USD 159,312 million in 2019, and it is expected to rise to USD 193,914 million by 2027 [1]. Besides the healthy characteristics of seafood, changes in the lifestyles of Western and Far-Eastern populations, as well as increasing disposable income, have been driving the constantly growing demand for innovative and more convenient seafood products [1].

With increasing attention to and interest in varied and healthy diets, the acquisition and inclusion of seafood products in the daily diet of millions of consumers is a growing trend [2]. Seafood is an essential source of macronutrients, such as fat, protein, and carbohydrates, and micronutrients, such as minerals and vitamins, and due to its high nutritious value, an ever-growing demand for affordable, easy-to-use, and ready-to-cook seafood products is driving innovation [2,3]. By virtue of these characteristics, the adoption of processed seafood as a convenient alternative to the traditional non-processed products has made these products more accessible and adaptable to the busy lifestyle typical in developed nations [1]. Because of this, the supply of healthy, ready-to-cook seafood products is no longer a luxury but a necessity. However, as a result of their nutritional characteristics and composition, seafood products are highly perishable foods. Product degradation, as well as economic pressures, result in large quantities of product waste [4]. The loss of large amounts of product culminates in financial losses and quality concerns for both the industry and the consumers [3,5]. Both financial expenses related to the waste of products and ecological concerns increase the need to develop alternative or additional preservation methods.

Currently, refrigeration and freezing storage are the most common forms of fish preservation [6]. Although the ambient temperature is one of the most critical parameters responsible for the proliferation of spoilage and pathogenic bacteria [7], refrigeration alone cannot provide long shelf life periods for fish [6]. Therefore, the storage of seafood under freezing temperatures (<−18 °C) is currently the only technology that guarantees its preservation for long periods. Although efficient at preserving the product and inhibiting microbial spoilage, storage at freezing temperatures also negatively impacts the sensory qualities of fish [6]. In addition, consumers tend to avoid freezing seafood products, much more than what is observed in meat products, since fish and other seafood are perceived to be of higher quality and demand more delicate care [8]. This notion of high-value products even leads to the rejection of discounted seafood products, as the freshness of these low-priced products is questioned by the consumers. Therefore, a delicate equilibrium between convenience, freshness, and price must be maintained to achieve a desirable product [8]. While these factors induce innovation and serve as an incentive for extending the shelf life of products, consumer safety is a matter of utmost consideration to regulatory agencies. Thus, governmental organizations regulate methods used to extend the shelf life of foodstuffs. For example, the European Union establishes requirements for all the steps in the food chain, from production to the addition of substances to preservation methods. As described in Regulation (EC) No. 178/2002 [9], legal guidelines are provided to ensure consumer access to a safe and healthy diet. European regulation presents these requirements, and the EU enforces them throughout the member states. This being said, any type of innovation in the production, processing, preservation, and transportation of food products must respect European legislation to be approved as a legal practice in the industry. The European Food Safety Authority (EFSA) is responsible for reviewing innovations and new health claims and giving scientific advice to the commission [10].

Since bacterial activity is the leading cause of seafood spoilage, preservative techniques must strive to induce natural and artificial forms of antimicrobial activity to control the spoilage microorganisms [11]. These techniques include biodegradable films and edible coatings, superchilling, irradiation, high-pressure processing (HPP), and biopreservation [10,12,13,14,15,16,17,18]. This review will focus on the latest advances in the application of alternative techniques of shelf life-extending protocols and their effect on the preservation of seafood products. It is intended to provide information on long-period shelf life induction techniques as an alternative to frozen storage.

## 2. Degradation and Spoilage of Seafood Products

A product is considered spoiled once its sensory alterations are so representative that it is no longer fit for human consumption [19]. In seafood, changes in organoleptic characteristics such as fishy, ammonia-like, or sweet odors; off-putting taste; and an overall unpleasant aspect develop rapidly during the spoilage process [20]. This spoilage can be induced by a variety of causes, from oxidative spoilage and autolytic enzymatic spoilage to microbial spoilage [5,21]. The nutritional value of the product also decreases during spoilage. This spoilage is impacted by the specific properties of the product, conditions of handling, and conditions of storage [22]. High concentrations of fat, protein, and moisture, in addition to low tissue stability, provide bacteria with an optimum medium for proliferation [5]. Along with degradation and loss of nutritional value of the product, consumer safety can also be affected by the proliferation of pathogenic organisms capable of either producing harmful metabolites or leading to bacterial diseases in humans [7].

The degradation of fish products by the microbial activity of specific spoilage organisms (SSOs) is the most concerning cause of product spoilage faced by the producers [11]. Specific species of spoilage organisms can vary depending on the origin of the product and the processing techniques employed during its handling and storage [23]. While fish muscle is sterile, the gills, skin, and gastrointestinal tract have significant microbial populations [21,24]. This microbiota varies depending on the environment in which the fish develops and lives [5], with increased numbers of mesophilic bacteria being observed in tropical water fish and increased numbers of psychrophilic bacteria being observed in cold water seafood [21]. Nonetheless, there is an over-representation of the phylum of Proteobacteria. Usually, genera of this phylum found in seafood products are *Pseudomonas*, *Shewanella*, *Acinetobacter*, *Aeromonas*, and *Photobacterium* [5]. While Gram-negative Proteobacteria are the most common bacteria found in seafood, Gram-positive bacteria of the genera *Micrococcus* and *Clostridium*, as well as lactic acid bacteria (LAB), can also be present in various quantities [19]. Microbial proliferation and metabolism of bacteria such as *Shewanella* spp., LAB, and *Photobacterium* spp. leads to the production of compounds such as trimethylamine, ammonia, hydrogen sulfide, methyl mercaptan, ethanol, and dimethyl-disulfide from the reduction of trimethylamine oxide, the metabolization of urea or deamination of amino acids, and the breakdown of sulfurous compounds [20,24,25]. In addition, the production of biogenic amines, such as cadaverine, putrescine, and histamine, can be induced by the decarboxylation of amino acids by bacteria such as *Shewanella putrefaciens* and *Enterobacteriaceae* such as *Hafnia alvei* and *Morganella morganii* [21,24]. Histamine and its precursor histidine are highly regulated compounds due to their activity in provoking allergic reactions in the consumer [26,27]. While many spoilage bacteria found in seafood are naturally present in the gastrointestinal tract, skin, and gills, Møretrø et al. [2] noted the presence of *Pseudomonas* spp. and *Shewanella* spp. in high quantities on equipment and zones of bleeding and short-time storage of several processing plants. The presence of high levels of bacteria in industrially processed fish highlights the necessity of strict hygiene standards during the production process to avoid contamination [2].

Furthermore, besides spoilage-inducing bacteria, the growth of pathogenic microorganisms in food products also demands significant attention. Seafood-associated illnesses have been linked to the presence of viruses (e.g., hepatitis A and noroviruses) [28], bacteria (e.g., *Vibrio* spp., *Salmonella* spp., *Listeria monocytogenes*) [29], and parasites (e.g., *Anisakis*) [30,31,32]. Due to its over-representation as the primary cause of foodborne disease, the control of pathogenic bacteria must be the primary focus in achieving food safety. *Vibrio* spp., *Clostridium* spp., *Salmonella* spp., *Shigella* spp., *L. monocytogenes*, *Staphylococcus aureus*, and *Escherichia coli* can be present in food products due to cross-contamination and proliferation of microorganisms from the gastrointestinal tract of the animals [33,34] and pollution of the environments in which the seafood is produced [30]. To guarantee that any product is appropriate for consumption, several methods, not dependent on sensorial observations, are used to evaluate the freshness of the seafood. These methods are based on bacterial count limits, such as those imposed by legislation (e.g., regulation 2073/2005 in the European Union) or recommended by, for example, the International Commission on Microbiological Specifications for Food (ICMSF) and the Food and Agriculture Organization (FAO), or indicators of metabolic activity of spoilage agents [21] such as total volatile basic nitrogen (TVB-N) [35], which is an indicator of the degradation of proteins and amines; thiobarbituric acid reactive substances (TBARS) [36], by-products of lipid oxidation; Trimethylamine N-oxide (TMAO) [37], an amine oxide which is a consequence of bacterial activity and results in strong “fishy” odors and cardiovascular events in humans; and peroxide value (PV) [38], an indicator of deterioration and oxidation of lipids. In addition, the development of new techniques to evaluate fish freshness, preferentially non-destructive, eco-friendly, less time-consuming, and more accessible than the conventional techniques, has been studied [3]. Techniques such as the evaluation of freshness through biosensor techniques [39,40], bionics methods [41,42], and spectroscopic technologies [43,44] have been developed; while these techniques are considered to be insufficient to detect the spoilage of food on their own, they show great potential when coupled with conventional methods [3].

**Figure 1:**
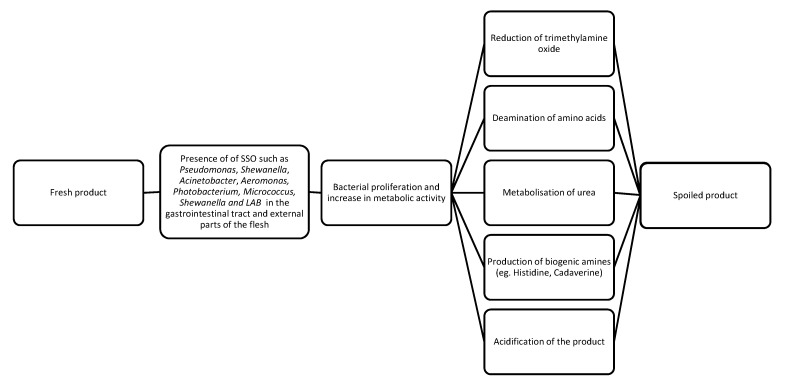
Schematic summary of the degradation and spoilage of seafood products.

Figure 1 summarizes the degradation and spoilage of seafood products.

## 3. Strategies for Preservation of Seafood Products

In this section, preservation techniques already in use or with the potential to be applied to seafood products are evaluated. A brief introduction to each technique is presented, and when possible, a compilation of recent studies regarding its application to fresh seafood products is presented. For each technique, the antimicrobial activity and impact on the hindrance of sensorial degradation are focused on. In addition, indicators of metabolic activity of spoilage organisms such as TVB-N, TBARS, TMAO, and PV are analyzed in cases where such data are available.

### 3.1. Biodegradable Films, Edible Coatings, and Natural Preservatives

The application of films or coatings composed of edible and biodegradable compounds allowing the extension of shelf life and freshness periods of seafood products has been the focus of several recent studies [45,46,47,48,49]. These films and coatings can retard the spoilage of the products by inhibiting bacterial proliferation and promoting a protective layer between the product and the environment, retaining the sensorial properties of fish, such as smell, texture, and flavor [10]. Films and coatings differ in the form of application to the matrix. While both techniques are composed of the same compounds, coatings are a specific type of film, which is applied directly on the matrix and is part of the final product, whilst films possess plastic-like properties and can be separated from the product [50]. Films, being thicker, can be used as biodegradable, chemically active alternatives to plastic packaging [50].

To achieve formulations with no safety concerns for the consumer, all components must be food-grade and safe to ingest. Should these requirements be followed, films and coatings can be applied to most food products with no threat to consumer health [51]. The use of natural and non-toxic compounds as coating and film materials permits the use of antimicrobials and preservatives while keeping the product free of synthetic additives, complying with consumer demands for safe and natural shelf life-extending technologies. By limiting interaction with the exterior and serving as a semi-permeable layer, films and coatings restrict gaseous transfers, water migration, solute movement, and bacterial respiration [50]. This protection results in the maintenance of sensorial characteristics for extended periods.

The application of natural coatings is an encouraging approach capable of answering the increasing demand for ready-to-cook products while maintaining a completely natural composition [52]. Because of their popularity as a promising innovation, numerous antimicrobial components have been incorporated into these edible coatings with positive results [10,53,54]. Edible coatings and biodegradable films usually consist of a solution of lipids and/or polymers, such as proteins and polysaccharides, with antimicrobial activity, such as chitosan, or capable of forming a structural matrix operating as a carrier of antimicrobial compounds, such as sodium alginate and carboxymethylcellulose [10,50,55]. Polysaccharide-based films and coatings, such as those consisting of cellulose, chitosan, and alginate, benefit from the abundance of such compounds in nature. In addition, these appear to be characteristically efficient in inhibiting gas transfers, limiting the impact of oxidation, dehydration, and overall sensory degradation [51]. While not all polysaccharide-based coatings have the same properties, their effects on food preservation seem to be generally beneficial [50]. Chitosan has been highlighted as a result of its antimicrobial properties [56,57]. Originating naturally only in fungi, such as those of the *Mucoraceae* family, it can also be manufactured through the deacetylation of chitin, one of the most abundant components of insect and crustacean exoskeletons [57,58]. In some cases, its application in edible films or coatings was proven to preserve foodstuff with excellent efficiency [59,60]. Such is observed by several authors, with the considerable extension of shelf life of fish fillets by the application of edible coatings in association with essential oils [46], phenolic acids [47], and propolis extracts [16]. Furthermore, besides their antimicrobial activity, chitosan-based solutions can incorporate functional compounds and, in doing so, increase the nutritional and functional value of food products [61]. However, chitosan is often soluble in acidic environments, thus limiting the potential use of this compound in the coating of food products [57]. Starches also show potential in retarding the degradation of seafood and extending shelf life. Due to their flexibility and adhesiveness properties, starch-based edible films and coatings appear to be efficient protective agents. As seen in the study of Korkmaz et al. [48], coatings mainly composed of starch of quinoa origin managed to reduce lipid oxidation, improving quality parameters of the products and ensuring these were maintained for more extended periods. Likewise, alginate, a polysaccharide extracted from seaweed [62], and cellulose [63] present viable solutions to the demand for polysaccharide-based coatings. While alginate and cellulose have no intrinsic antibacterial activity, Baek et al. [62] and Raeisi et al. [63] reported significant increases in shelf life, control of gaseous transfers between the food matrix and the environment, and bacterial inhibition of alginate and cellulose coatings, respectively, in seafood. This reduction in sensorial and quality decay, resulting from the protection of the product, demonstrates the physical impact of polysaccharide-based coatings in seafood.

Lipids, being predominantly hydrophobic compounds, impose barriers to water migration and, therefore, prevent drip loss and shrinkage of food products [64]. Lipids, such as wax and paraffin, can be used as the main constituents of coatings and films, resulting in considerably thicker films, or associated with polysaccharide or protein coatings [50]. These associations are known to result in strongly hydrophobic, cohesiveness-strengthening edible coatings [50]. Wax- and paraffin-based coatings have been used for almost a century for the protection of fruits and vegetables [64]. Due to the brittle and thick texture they add to the treated product and their lack of structural cohesiveness, lipid coatings alone are rare, and these compounds are more commonly associated with protein- or polysaccharide-based coatings [64]. Propolis is a mixture of beeswax and other resins collected by honeybees and is commonly used in alternative medicine products due to its reported functional properties [65]. Extracts of propolis can be added to other coatings such as chitosan-based coatings to increase their antimicrobial activity, as observed in the study of Ebadi et al. [16]. Extract composition may vary according to geography and surrounding flora; however, strong antibacterial activity is commonly found as a characteristic of propolis extracts [66].

Lastly, protein-based films and coatings manage to easily adhere to the surface of food matrices due to their hydrophilic properties [67]. These agents present significant hindrances to gaseous transfers but might be somewhat permeable to water. Protein coatings have the advantage of being naturally nutritive and, due to their functional properties, can increase product value [67]. While capable of inhibiting bacterial proliferation and degradation of seafood, the efficiency of these protein-based coatings appears to be improved with the addition of essential oils. For example, improvements in lipid oxidation retardation, TVB-N value reduction, and bacterial growth inhibition in seafood were observed in zein [68], fish gelatin [69], and whey-based coatings [67], with the addition of *Pimpinella affinis*, oregano, and cinnamon essential oils, respectively.

Similarly, collagen-based coatings doubled the shelf life of mackerel fillets when in association with essential oils, as observed in the study of Hu et al. [70]. Specifically for the coating of seafood, since fish gelatin and collagen are also by-products of fish processing, the use of these materials and fish scale collagen composition and conformation results in improved nutritional values and tensile properties compared to collagen of different sources [70]. The use of these seafood processing by-products is of particular interest when a protein-based edible coating is desired [70].

Besides films and coatings, the application of several plant-based compounds as additives in food products has been increasingly regarded as a safe alternative to the conventional synthetic compounds used to preserve food products and extend their shelf life [71]. Due to their antimicrobial activity and functional properties, various essential oils and compounds such as limonene, thymol, oleuropein, and carvacrol have been the focus of several studies [54,72,73]. By adding essential oils and chemicals with antimicrobial properties and their constituents to the formulation of edible coatings, significant health benefits can be obtained [53]. For example, thyme essential oil, a safe and natural essential oil extracted from *Thymus vulgaris*, has been regarded as an alternative to the commonly used preservatives and shelf life-extending agents due to its intense antimicrobial activity [66]. Due to its high concentrations of thymol, a potent antibacterial component, thyme essential oil has been the subject of extended studies regarding their application in food products [53,74]. Ozogul et al. [74] reported that the antibacterial activity of thyme essential oil was remarkably efficient against foodborne pathogenic bacteria and fish spoilage bacteria; its minimal inhibitory concentration was lower than that observed for tetracycline, streptomycin, and neomycin. Memar et al. [71] detected identical antibacterial and antifungal activity in carvacrol. This may result from the similarity of conformation and origin of these compounds since both are extracted from the *Lamiaceae* family of plants [75]. Antimicrobial, antioxidant, anti-inflammatory, cardioprotective, and neuroprotective properties have been reported for carvacrol [71]. Limonene has also been commonly used in the food industry due to its aromatic and flavor-inducing properties [11]. Limonene is an aromatic compound present in various natural essential oils, specifically those extracted from citrus fruits. It is regarded as a safe compound, and its application in food products is regulated in the Union List of Flavourings and Source Materials of the European Union (EU No. 872/2012, 2012) [76]. With antimicrobial, antioxidant, and anti-inflammatory properties, limonene application in food products has been the target of several studies [11,77].

Essential oils are also known to yield strong smells and flavors due to their composition rich in characteristically volatile compounds [66]. Thus, the impact of essential oils and other aromatic compounds on the sensorial characteristics of food products can result in off-putting attributes for the consumer. Therefore, their use should be kept to the minimum necessary.

A variety of recent studies found in the literature about different film and coating agents used in seafood products is shown in Table 1.

### 3.2. Superchilling

Superchilling (SC) or, in more industrial terms, subchilling [13], is the procedure through which a certain percentage of water in food, around 5–30%, is cooled below the freezing point [13]. In this process, the temperature of the product is reduced to 1–2 °C below its freezing point. At these temperatures, some water solidifies into ice at the surface and slowly starts migrating to the core of the product [84]. Since traditional techniques for preserving these products consist of storage at refrigeration temperatures (0 to 4 °C) or frozen storage (−40 to −18 °C), superchilling employs temperatures below what would be regarded as refrigeration, but considerably higher than freezing temperatures [85]. While the interior reaches temperatures lower than the freezing point, the initial superficial ice coating disappears, leaving the product to appear fresh or chilled [84]. The product is then stored for an extended period under these temperatures, preserving its characteristics. If the right conditions are applied, differences between a superchilled and a fresh, non-treated product in texture, color, and overall appearance are mostly undetectable. This is important to maintain consumer agreeableness and interest [84]. However, the impact of superchilling on quality parameters depends on the intensity of the technique, temperatures applied, and amount of water cooled below the freezing point [85]. Increases in product drip loss, for example, appear to be related to the high percentage of freezing water in the product [85]. Nonetheless, superchilling leads to a considerably longer shelf life than refrigeration [85].

While the product maintains fresh characteristics, its iced interior contributes to the stability of quality parameters throughout prolonged periods. Thus, this icy core inhibits the growth of undesirable microorganisms, such as H_2_S-producing bacteria, and the consequent spoilage of the product [13]. Such an effect was observed in the study of Eliasson et al. [13], where superchilling impacted not only H_2_S-producing bacteria but also inhibited total viable growth, prolonging chemical and microbial quality characteristics of Atlantic cod and extending the freshness period. Superchilling improves, therefore, the shelf life of the product. However, since different products have different compositions and structures, this process requires optimization for every product to which the technique is applied [84]. The application of temperatures lower than the optimal 1–2 °C can lead to the formation of sizeable unwanted ice crystals, which in turn results in the physical degradation of the product. In contrast, not low enough temperatures can fail to inhibit bacterial proliferation and subsequent microbial spoilage [84].

The application of superchilling combined with other techniques has been the subject of various studies [6,86,87,88]. This effort to synergize additional shelf life-extending approaches with superchilling resulted in the combination of edible coatings, essential oils, ice glazing, and modified atmosphere with superchilling. For example, in the study of Ye et al. [88], the combination of superchilling treatment and HPP resulted in an extension of shelf life to over 3 times what was observed in control samples, causing a considerably high drip loss. He et al. [6] demonstrated that an ice glazing enriched with clove essential oil associated with the superchilling technique managed to preserve sensorial properties by inhibiting bacterial growth and regulating oxidation, as indicated by the low PV and TBARS values observed in treated samples. Typically, ice glazing involves the formation of an icy barrier that coats the product, protecting it. This is achieved by the submersion of the product in a cold, nearly frozen solution [6]. The solution used is typically water, but with the progressively increasing knowledge of the preservative effectiveness of essential oils and vegetal extracts, new formulations have been tried [6]. With the addition of natural antimicrobial and antioxidant essential oils to the ice-glazing solution, not only liquid and gaseous transfers are controlled, but the action of microorganisms is actively inhibited, resulting in an efficient extension of the shelf life of the product [6]. In addition, several authors combined superchilling with modified atmosphere packaging (MAP). MAP consists of packaging food products in controlled atmospheres of one or multiple gases [89]. Usually, carbon dioxide, nitrogen, and oxygen are the main constituents of these modified atmospheres [89]. Higher than normal concentrations of any of these gases result in the inhibition of bacterial growth. By itself, this technique has shown significant potential in delaying the deterioration of seafood [89]. However, its combination with other techniques has also been studied [90,91,92,93,94,95,96], allowing the extension of shelf life even longer than observed in samples stored exclusively in MAP [86,94,95,97]. Skirnisdóttir et al. [86] applied a chitosan coating to this MAP–SC combination and reported not only the inhibition of bacterial growth but also the reduction in total viable counts as a result of chitosan antibacterial activity.

Superchilling appears as an alternative to other thermal techniques for the preservation of seafood. Alongside this, by provoking some freezing of the water present in the product, superchilling reduces the need to add ice to preserve seafood during short-range transportation and temporary storage [85]. Therefore, reducing energy consumption and transport weight results in less environmental impact due to these activities [85].

Some recent studies focused on the effect of superchilling on the quality and preservation of seafood are presented in Table 2.

### 3.3. Ozonation

Although the use of ozone in the medical field dates back to the 19th century, its use in food production has gained particular relevance in recent years [101]. Mainly being applied as a disinfectant and decontaminant of water [102], for drinking purposes, the use of this technique directly and indirectly in the processing of solid foods has more recently gathered interest [102,103,104,105,106]. With the introduction of cheaper and more convenient ozone generators, this technique is becoming progressively more accessible to be applied in an industrial context [101]. Ozone is a molecule composed of three oxygen atoms displayed in a bent, or “V”, geometry [107], with intense oxidative activity and, therefore, disinfectant and antimicrobial efficiency [101]. This compound interacts with cell components, such as fatty acids, proteins, and amino acids, oxidizing them and consequentially increasing cell permeability [101]. If sufficient doses and treatment time are applied, ozone activity eventually results in the lysis of cells.

Ozone antimicrobial activity has been shown to be highly efficient in inhibiting the growth of and destroying several microorganisms [108], such as Gram-positive and Gram-negative bacteria [109], yeasts, molds, and viruses [110,111]. Ozonation affects not only vegetative cells, but also bacterial spores by reducing their ability to germinate [109]. Since the presence of these organisms is a major cause of seafood spoilage [11], the versatility of ozone in negating the effects of a wide spectrum of spoilage-inducing organisms makes this technique an efficient preservative of sensorial properties and quality characteristics of seafood [108]. Ozonation can be directly applied, to food products, dissolved in water, through the washing, dipping, or spraying with ozonated water [110], and in a gaseous state [102,112] or indirectly applied by including this compound in ice used to keep the product at low temperatures [103,104,113].

While ozonation has been used for decades, and its application in various solid products has been a fairly common practice, few studies on the impact of this technique on seafood exist. Nonetheless, in the study of Chen et al. [113], a combination of ozone–slurry ice doubled the shelf life of fish by maintaining TVB-N values throughout storage, inhibiting bacterial growth and preserving texture, appearance, and odor for extensive periods compared to control samples. Similar finds were observed in the studies by Campos et al. [103,104] where both turbot and sardine shelf life periods were considerably extended by incorporating ozone in slurry ice refrigeration systems. In addition, Gonçalves et al. [96] and Okpala [114] reported improved quality attributes and preservation of shrimp resulting from ozonation with water dissolved and gaseous ozone, respectively. Identical results were observed in the study of Nerantzaki et al. [106] after dipping rainbow trout in ozonated water. Additionally, in the study of Gelman et al. [105], tilapia fish were subject to ozonation while alive by being placed in 100-L water tanks with 6 ppm ozone, which resulted in up to 3 days of shelf life-extension in the flesh from these fishes. Therefore, ozonation appears to efficiently preserve seafood products regardless of the application method used. Although ozonation acts by oxidizing cell components and some lipid oxidation would be expected of its activity, Chen et al. [113] showed that if used in the correct doses, this technique inhibits the rate of lipid oxidation of seafood by regulating microbial metabolism in seafood products [113], without causing any significant oxidative damage to the product. However, Crapo et al. [111], although detecting reductions in total viable bacteria in raw salmon after washing with ozonated water, also observed increased “rancidity” of the product.

Besides reducing total bacterial counts, and consequentially bacterial activity, ozonation of seafood products can also be used as a decontaminant against pathogens [112]. Feng et al. [115] reported the inactivation of *Vibrio parahaemolyticus*, a severe foodborne pathogen, resulting from the activity of ozonated water. Expression of most genes was observed, and the cell membrane was degraded to the point of inducing significant permeability. Similarly, Crapo et al. [111] detected inhibition of *Listeria innocua* identical to what was observed in samples treated with chlorine. Alongside pathogen decontamination, in the study of Louppis et al. [116], ozonation of mussels contaminated with diarrhetic shellfish toxins considerably reduced the presence of these toxins. These results indicate that this technique has great potential in ensuring long-lasting and safe seafood products.

The association of this technique with other preservation methods, such as MAP [96], suggests that considerable shelf life-extensions of seafood might be achieved from the synergy between the techniques.

### 3.4. Irradiation Techniques

The use of ionizing radiation to ensure better microbiological characteristics of food products has been a common practice [117]. Since the irradiation with electromagnetic waves of high frequency on cells results in considerable damage to DNA [118], the discharge of sufficiently high doses of gamma- and X-rays, as well as electron beams (EBI), onto foods reduces bacterial viability and proliferation [119]. This antibacterial activity can induce an efficient reduction in foodborne illnesses and biologically mediated spoilage of foods [119]. Bacterial inhibition is dependent on the food matrix, bacterial target, and radiation dose applied [117]. Low radiation doses, 0.5 to 3 kGy (kilogray), are tested quantities regarded as safe and efficient in prolonging the shelf life of seafood while maintaining the nutritional values of these products [120]. Higher radiation doses, while displaying increased antibacterial activity, might lead to unwanted changes in hedonic characteristics such as texture, cohesiveness, and resilience of seafood [117,121]. As observed in the study of Yu et al. [122], deterioration of texture and cohesiveness was evidentially associated with increased radiation intensity. However, as reported by Pan et al. [18], only radiation doses greater than 10 kGy could eliminate *Psychrobacter* cells from *Portunus trituberculatus*, and only with doses greater than 6 kGy did TVB-N values remain stable and not increase throughout storage. Since seafood products are generally stored in refrigerated conditions, the elimination or inhibition of psychrophilic bacteria is of utmost importance [117]. Pan et al. [18] also found a correlation between the intensity of radiation applied and the characterization and distribution of the microbiota in the product, indicating that tolerance to radiation varies according to the bacterial species. This variability is a consequence of the capability of nucleic acid repair activity by each organism, with those having more competent repair enzymes also being more resistant to radiation effects [123].

Since this technique also affects fungal cells and spores, this treatment prevents the spread of yeasts and molds [117]. Adding to this, bacterial spores, which are considerably more resistant than vegetative cells and can be found to survive some thermal treatments, are also negatively affected by the impact of electromagnetic radiation [117].

While maintaining better acceptability values, irradiation techniques have been known to increase thiobarbituric acid (TBA) concentrations in seafood [117]. This compound and its elevated concentration can lead to changes in color and taste. Such was observed by Yang et al. [120] where TVB-N, acceptability, and overall shelf life values were improved through this technique, but TBARS values increased beyond what was observed in control samples. This is a result of an increase in lipid oxidation and is particularly relevant in highly fatty seafood products [117]. Regardless of this, the general acceptability of irradiated products appears to be systematically superior to that of non-irradiated ones.

While being efficient in extending the shelf life of seafood, some unwanted and alarming alterations to the product should be considered. From increased permeability of the cell to modifications in the structure of proteins, changes in the product can be the result of absorption of energy during treatments with these high-frequency electromagnetic waves [123]. Harrell et al. [123] warn that some detrimental effects might result from the consumption of irradiated products since animal trials detected considerable amounts of radioactivity in organs of animals fed with irradiated foods. Mutations in rats, such as nutritional muscular dystrophy and increased mortality, because of internal hemorrhages, were observed as being caused by the impact of radiation on vitamins K and E, which in turn would lead to these being less absorbable by the consumers [123,124]. In addition, some results indicate correlations between the irradiation of food and increased amounts of carcinogenic compounds [123,125]. Irradiation is, therefore, a polarizing subject, with Tritsch [125] even suggesting that should this technique become “widespread, it will take four to five decades to show statistically significant increases in cancer incidence”. Additionally, the construction of processing plants with irradiation capabilities is significantly expensive; therefore, this type of processing plant is not widely distributed worldwide [117].

As mentioned above, some authors studied the impact of irradiation techniques on the quality and preservation of seafood. A compilation of these studies and the main results are presented in Table 3.

### 3.5. High-Pressure Processing and Hyperbaric Storage

High-pressure processing (HPP) has been regarded as an adequate alternative to the more conventional, thermal, methods of food preservation [128]. It acts by processing the product at high pressures, suppressing bacterial growth and, consequently, extending shelf life expectancy [129].

With industrial equipment capable of reaching pressures up to 1000 MPa, most enzymes and microorganisms are efficiently eliminated or inactivated [130,131]. This antimicrobial activity is a consequence of the impact that high pressure has on the denaturation of proteins and the destabilization of cell walls and cell membranes. HPP has also been found to affect DNA and ribosomal activity [131]. Besides being capable of inhibiting the bacterial growth of vegetative cells, HPP is also known to destroy bacterial and fungal spores, yeasts, and molds [131]. Microbial susceptibility to this treatment is, however, dependent on the microorganism’s capacity to resist and repair damages caused by pressure [132]. Bacterial structure, for example, seems to play an essential part in defining the intensity needed to inactivate a specific species, with Gram-positive bacteria showing increased tolerance to pressure. This is a consequence of the increased cell wall complexity these bacteria possess [132]. Nonetheless, pressures over 600 MPa destroy most vegetative cells. Bacterial spores are, however, considerably more resistant than vegetative cells; spores of *Bacillus cereus* and *Clostridium botulinum* are only efficiently destroyed with pressures over 1000 MPa [130]. While spores seem resistant to greater pressures than those produced by industrial equipment, Modugno et al. [130] reported that pressures of 500 MPa induced germination of “a large proportion” of *Bacillus subtilis* spores, leading to the inactivation of at least some of these cells.

Since the impact of these extremely high pressures targets exclusively non-covalent bonds of molecules, bacterial inhibition ensues with a little negative impact on nutrition and the overall quality of the product [132]. Therefore, the non-thermal characteristic of HPP allows for a reduction in bacterial counts while limiting organoleptic changes that might occur when using more traditional methods such as freezing [133]. This depends, however, on the pressures used since higher pressures can leave a “cooked” appearance and considerable textural changes in the product [133]. Besides some modifications to the sensorial characteristics of seafood, various authors detected increases in lipid oxidation and TBARS [129,133,134]. Because this indicates deterioration and low quality, it is expected that sufficiently high pressures can provide unacceptable amounts of these compounds in seafood [135]. This increase in lipid oxidation values appears to be connected to the characteristics of the product, with high-fat fish being more susceptible than low-fat seafood. This was observed in the studies of Rode et al. [129] and Arnaud et al. [133], where the authors detected an increase in malondialdehyde (MDA) values, another standard indicator of lipid oxidation, in mackerel and salmon but not in cod. In addition, Rode et al. [15] and Giannoglou et al. [136] found no indication of excessive or increased lipid oxidation in cod or seabass.

Because different seafood species have different compositions, there is no standard value of pressure needed to decontaminate the product which is applicable to all products [129,133]. Therefore, it is imperative to study the impact of HPP in each food matrix before use. If the pressures applied are not exaggerated, high-pressure processing and its active intervention in physical and chemical degradation of the fish product helps in the maintenance of reasonable values of texture, flavor, odor, and appearance [129]. This, associated with the strong preservative activity of HPP, allows the conservation of good characteristics and microbiological safety of seafood. As reported by Rode et al. [129], cod, salmon, and mackerel shelf lives were increased by up to 11, 10, and 4 days, respectively, when treated at 200 MPa of pressure [129]. These results show the potential of HPP technology in the considerable extension of food products’ shelf life.

HPP activity in the inactivation of enzymes and denaturation of proteins could also be helpful in controlling the allergenicity of foods [14]. The inhibition of parvalbumin, the primary allergen in fish, by applying pressure to the product could help millions of people affected by such conditions [14]. This process shows potential in removing entirely or, at least, reducing the presence of allergens in food. Its efficiency depends on the type of product, and further research is needed for its application in fish products [14].

While showing promising results in maintaining texture, flavor, and appearance, HPP is responsible for the denaturation of proteins and color changes in some fish products. Depending on the pressure applied and the characteristics of the product, the texture is affected as well [137]. Even though HPP might not be used as a substitute for conventional preservation techniques, its activity in microorganism destruction and enzymatic inactivation can improve food shelf life and quality when coupled with other, more traditional methods [137]. The impact of HPP on the quality and preservation of seafood, found in some published studies, is summarized in Table 4.

Sharing the same principle as HPP, in which high pressures are applied to inhibit bacterial growth, the storage of foods at considerably high pressures is also regarded as a valid alternative to the conventional preservation and storage methods. However, contrastingly to what is observed in HPP, pressures applied in hyperbaric storage (HS) are usually lower than 200 MPa [139], and these pressures are maintained for long periods, from hours to months [139]. By maintaining such conditions and not needing a constant energy flux to lower the storage temperature, as is typical in refrigerated or freezing storage, HS appears to be more energetically effective [140]. This happens because this technique only needs the energy to reach the desired pressure, reducing its energy consumption after this point [140].

The use of HS at room temperature in seafood has resulted in different outcomes; Fidalgo et al. [141] observed the maintenance of fresh characteristics in Atlantic salmon (*Salmo salar*) under hyperbaric storage long after the refrigerated controls began showing signs of deterioration, but Moreira et al. [140] did not detect differences between both storage techniques. Nonetheless, Moreira et al. [140] praised hyperbaric storage for its low energy consumption and reduced carbon footprint compared to refrigerated storage. Fidalgo et al. [4] also observed significant bacterial count reductions in salmon immediately after pressurizing meat up to 75 MPa, extending shelf life from 3 days, in refrigeration, to over 25 days. However, as observed in samples treated by HPP, some increase in lipid oxidation also occurred due to this technique. Similarly, Otero et al. [142] did not detect bacterial growth in samples of mackerel (*Scomber scombrus*) stored at 50 MPa during the first 15 days, in which total viable counts remained near, or below, what was observed in the control on the first day. In addition, sensorial characteristics and physical properties were superior to those observed in refrigerated samples [142]. Regarding physical stability, Fidalgo et al. [143] observed clear improvements in maintaining muscular structure and conserving drip loss and water holding capacity throughout storage. Chemically, samples of Atlantic salmon remained closer to the initial samples when compared to refrigerated samples, having alcohol and aldehyde concentrations that remained stable for 15 days, indicating reduced microbial activity [143].

Therefore, hyperbaric storage represents an interesting alternative for industrial storage of seafood products, enabling the preservation for more extended periods while reducing energetic costs and ecological impact. Nevertheless, industrial equipment for hyperbaric storage in the food sector still needs to be commercialized.

### 3.6. Biopreservation

Biopreservation techniques consist of the use of microorganisms and products of microbial origin to preserve and control bacterial proliferation in food products [144]. Produced by lactic acid bacteria (LAB), bacteriocins, peptides with antibacterial effects, have shown efficiency in killing or inhibiting the proliferation of undesirable bacteria in food products [145]. Therefore, LAB and their bacteriocins can be applied to food products with the aim to preserve and consequently extend seafood shelf life [144,146]. The use of these natural antimicrobials has been the focus of various studies searching for solutions to the current demands for alternative decontamination methods [147,148,149,150]. For example, López de Lacey et al. [151] inoculated *Lacticaseibacillus paracasei* in hake, prolonging its shelf life for over a week and lowering total viable counts by reducing spoilage bacteria impact on the degradation of the product. Inoculation of food with LAB cultures or bacteriocins can also be used to target specific bacteria such as Helicobacter pylori, which was significantly inhibited by the presence and activity of *Limosilactobacillus reuteri*, thus reducing the impact of this pathogen and the stomach pathologies provoked by its infection [152]. Tomé et al. [153] also showed inhibition of *Listeria* spp. growth in vacuum-packaged cold-smoked salmon as a result of *Enterococcus faecium* activity in the food matrix. In addition to their antimicrobial activity, LAB can also be used as functional agents, improving the antioxidative characteristics of fish and decreasing the presence of free radicals in the product [152]. Their ability to produce various vitamins shows that great value can be added to the product by applying microorganisms such as *Lb. reuteri* in food products [152].

Since LAB are known to produce lactic acid, their metabolic activity can also result in undesirable changes in the product since acidification can lead to structural, sensorial, and nutritional degradation [154,155]. This acidification can even lead to decreases in the acceptability of seafood and loss of product. Such an adverse effect of inoculated LAB was recently observed by Wiernasz et al. [156], where *Aerococcus viridans*, *Lactococcus piscium*, and *Leuconostoc gelidum* reduced acceptability, sensory scores, and shelf life of salmon. Because of this, the optimization of biopreservation processes using LAB must consider the species and their level to achieve the desired effects [17]. Likewise, it is also imperative to select species and strains capable of producing metabolites in a considerable quantity, and for these metabolites, whether they are of low molecular weight, such as peroxide (H_2_O_2_) and carbon dioxide (CO_2_), or of high molecular weight, such as bacteriocins, to have a wide range of effective inhibition of unwanted pathogens and spoilage organisms [17,157].

Along with bacteria and bacterial by-products, the use of bacterial-specific viruses, bacteriophages, can reduce microbial counts in food matrices [147]. Their stability during storage, bacterial specificity, and self-replication capabilities provide antibacterial activity in a generally safe and biological manner [158]. Bacteriophages, or more colloquially, phages, are bacteria-specific viruses capable of infecting and destroying bacterial cells [159]. Bacteriophages already play an important role in ecological balance since they limit the proliferation of specific bacteria in a given environment [160]. With the constant advances in biotechnology and virology, the application of bacteriophages as food safety agents has received increased attention [161,162,163]. While some doubts regarding the impact of phages on the immune system and the presence of endotoxins in phage cocktails still exist [159], bacteriophages are generally regarded as safe due to their specificity for bacterial hosts [147].

Only lytic bacteriophages are suitable for food applications since these, through phage lytic enzymes, check bacterial proliferation by damaging the cell wall of the target [160]. These act through the hydrolyzation of the peptidoglycan, compromising the integrity of the cell wall and causing hypotonic lysis [164].

Phages used in food preservation show potential, not only due to their efficiency in bacterial inhibition but also due to being harmless to eukaryotic organisms. Because each strain of bacteriophage is infectious to a limited group of hosts, the application of bacteriophages in food products can, theoretically, inhibit unwanted bacteria while having no undesirable effect on human or animal commensal microbiota [160]. Through bacteriophage-dependent methods, specific pathogens can also be targeted and destroyed while preserving non-pathogenic bacteria [158]. Zulkarneev et al. [165] and Li et al. [161] reported reductions in total viable counts and an increase in the shelf life of seafood products when bacteriophages were added to the matrix. While not observing total bacterial count decay after treatment with phages, Hernández et al. [163] noticed inhibitions of up to 90% of bacterial cells of the *Serratia* genus. This result shows the strong and efficient impact of phages in a target bacterial community. This selective inhibition was especially relevant in the study of Baños et al. [147], where *L. monocytogenes* growth in seafood products was targeted and inhibition was achieved using specific bacteriophages. Since this microorganism is regarded as one of the major agents of foodborne illnesses [166], control of pathogenic bacteria such as *L. monocytogenes* is a valid achievement, even if no inhibition of total viable counts or shelf life-extension is achieved.

While the application of biopreservation techniques does not guarantee the inhibition of total viable bacterial cells, as seen in the study of Yamaki et al. [162], results show that the effective inhibition of a specifically targeted organism can be accomplished [161,162,163]. In addition to this, this method is fairly simple to apply to seafood, requiring only the treatment of the food matrix with inoculated solutions by means of pulverization, spraying, or dipping [147,153,154]. All these results suggest that biopreservation techniques are a promising substitute for and practical addition to the more conventional treatment methods of fish products [147].

A compilation of some recently published studies regarding the preservation of seafood products and the extension of shelf life periods can be observed in Table 5.

### 3.7. Comparative Analysis of Alternative Shelf Life-Extending Protocols

As seen above, all techniques appear to improve on refrigeration, extending shelf life, maintaining organoleptic characteristics, and reducing bacterial loads. However, the application of these techniques demands caution. High treatment intensity might cause product degradation, while low treatment intensity can fail to prevent bacterial proliferation and the resulting spoilage. In addition, financial costs must be taken into consideration when selecting the appropriate technique. Table 6 summarizes the main advantages and disadvantages of each technique mentioned above.

## 4. Conclusions

All the techniques mentioned appear to be potentially beneficial in ensuring a longer shelf life for seafood products. Not only can economic concerns be resolved through the investment in new technologies for the processing of this type of food product, but also improvements in food quality, food safety and ecological balance can be achieved. Possibilities to improve the stability and value of seafood, and by consequence, its access to more households, are vast.

Due to the enormous variety of organisms included in the term “seafood”, extensive studies must be performed before choosing the appropriate techniques. Microbiological, physical, chemical, sensorial, and economic parameters are just some of the parameters of which consideration is of utmost importance when changes to the processing of a food product are implemented. While shelf life-extension can be achieved by each of the techniques analyzed here, changes to the product might result in negative feedback from consumers.

The application of biodegradable and edible coatings can induce unwanted sensorial attributes, despite showing impact in retarding physical, chemical, and microbiological degradation. Changes in color, flavor, smell, and texture might be wrongly perceived as indicators of bad quality of seafood, regardless of the increase in nutritional value that the addition of some of these compounds might provide. In the same manner, the physical impact of superchilling might cause organoleptic alterations, especially when the process is not optimized for the specific product. In addition, to our knowledge, no apparent certainties on the public acceptability of superchilled products exist. It is unknown whether consumers’ expectations and willingness to pay for superchilled products are similar to those for fresh products or more akin to those for frozen products. Regarding irradiation and high-pressure techniques, both can alter the sensorial properties of the product if enough radiation or pressure is applied. The “cooked” appearance of over-pressurized products might reduce consumer interest in such products, even if freshness and shelf life periods are extended. While hyperbaric storage’s initial cost is significant, the energy-efficient property of this technique, associated with its eco-friendly characteristic, might be beneficial to producers and consumers. The unknown impact of radiation of extremely short wavelengths on food and consequentially on human health raises concerns regarding the safety of such products. Lastly, the application of LAB as biopreservative agents and their antimicrobial activity show considerable influence in extending shelf life. The production of safe antimicrobial compounds appears to retard bacterial proliferation and spoilage. However, the concentration, species, and strain used must be carefully selected since changes in pH might occur. On the other hand, bacteriophages can be chosen to target specific unwanted pathogens and/or spoilage organisms.

As seen, to achieve any significant shelf life-extension, any technique needs to be employed in higher than recommended doses, resulting in sensorial alterations or decaying nutritional value. Therefore, further studies focusing on the synergy of multiple methods in low amounts or intensities should be performed. We believe that, while none of the studied techniques can, by itself, present a solid alternative to replace the freezing of seafood products as the primary long-period preservative method, the introduction of these techniques as additional treatments can improve the preservative action of refrigeration.

## Figures and Tables

**Table 1:** Compilation of studies regarding film and coating agents for seafood products.

Compound	Additional Treatment	Species Tested	Results	Reference
Chitosan coating	Aspartic acid	Channel catfish (*Ictalurus punctatus*)	2 log cycles of reduction after 6 days. Regulation of pH and TVB-N values.	[45]
Chitosan coating	Whey protein and tarragon essential oil	Talang queenfish (*Scomberoides commersonnianus*)	Extension of TVB-N values under 30 mg/100 g from 8 to 16 days. pH changes contained. Over 2 log cycles of psychrotrophic and mesophilic bacteria reduction after 8 days.	[46]
Chitosan coating	Gallic acid	Horse mackerel (*Trachurus* *trachurus*)	4 days of extension of shelf life when nanoparticles and gallic acid were used. Regulation of pH and TVB-N values. Total inhibition of H_2_S-producing microorganisms.	[47]
Chitosan coating	Propolis extract	Japanese threadfin bream (*Nemipterus japonicus*)	Reduced lipid oxidation. Reduced TVB-N and pH values. Over 10 days of extension of shelf life. Improved sensorial characteristics.	[16]
Sodium alginate coating	*Zataria multiflora* Boiss essential oil	Trout *	Inhibition of total viable bacteria, total psychrophilic bacteria, hydrogen sulfide producing bacteria, and *Enterobacteriaceae.*	[78]
Furcellaran film	Green tea extract and synthetized selenium nanoparticles	Common carp (*Cyprinus carpio*)	Enhanced antimicrobial activity against *E. coli*, *S. aureus*, and MRSA. Great antioxidant activity.	[79]
Chitosan coating	Pomegranate peel extract	Nile tilapia (*Oreochromis niloticu*)	Inhibition of *Enterobacteriaceae,* coliform bacteria, *Salmonella* spp., *E. coli,* yeast and mold, and *Staphylococcus aureus* growth to undetectable levels. Control of TVB-N values under acceptable limits. Shelf life extension from <15 to >30 days. Preservation of sensorial characteristics for over 30 days.	[80]
Chitosan coating	Clove essential oil and kojic acid	White prawn shrimp (*Litopenaeus vannamei*)	Over 3 log cycles of total aerobic bacteria growth inhibition. Shelf life extension. Reduced TVB-N and pH increase. Preservation of sensorial characteristics. Reduced weight loss.	[81]
Sodium alginate and chitosan coating	Grapefruit seed extract	White prawn shrimp (*Litopenaeus vannamei*)	Extension of TVB-N values under acceptable limits from 8 to 12 days. Improved sensorial characteristics. Inhibition of psychrophilic and mesophilic bacteria. Reduced melanosis.	[82]
Sodium alginate coating	Grapefruit seed extract	Shrimp *	Reduced weight loss. Extension of TVB-N values under acceptable limits from 4 to over 8 days. Delay in chemical decay. Reduced melanosis. Enhanced overall acceptability.	[62]
Quinoa starch film	-	Rainbow trout (*Oncorhynchus mykiss*)	Chemical and biological protective effect. Resulted in slight but significant inhibition of bacterial growth and chemical decay.	[48]
Pectin/chitosan coating	Tarragon essential oil (*Artemisia dracunculus*)	Narrow-barred Spanish mackerel (*Scomberomorus commerson*)	Significant reduction in lipid oxidation. Lower bacterial counts. Reduced TVB-N and TBARS values. Extension of shelf life from 8 to over 16 days of storage.	[49]
Pectin coating	Gallic acid	Japanese sea bass (*Lateolabrax japonicas*)	Regulation of TVB-N and pH values. Some acidification of the matrix was observed. Considerable reduction in TBARS values. Sensorial characteristics remained acceptable for at least 5 days longer.	[83]
Carboxymethyl cellulose coating	*Zataria multiflora* Boiss essential oil and grape seed extract	Rainbow trout (*Oncorhynchus mykiss*)	Better microbial and sensorial scores in treated samples. Organoleptic properties remained acceptable through more extended periods of storage. Decrease in lactic acid bacteria and pseudomonas counts. Regulation of TVB-N increase.	[63]

Legend: * no binomial classification of species provided by the study.

**Table 2:** Compilation of studies on the effect of superchilling on the quality and preservation of seafood.

Additional Treatment	Storage Conditions (°C)	Species Tested	Results	Reference
-	−1.7	Atlantic salmon (*Salmo salar*)	Significant decrease in liquid loss after 1 day of superchilled storage. No significant differences after this point.	[98]
-	−1	Atlantic cod (*Gadus morhua*)	Extension of 2–4 days of freshness period and 3 days of shelf life. Lower microbial growth, H_2_S-producing bacteria, and total volatile basic nitrogen in superchilled samples.	[13]
-	−2	Peled (*Coregonus peled*)	Lower collagen degradation and extended texture retention period in superchilled samples. Colony-forming units per gram below FAO standard in superchilled samples after 6 days.	[99]
Cryoprotectants	−1; −3; −3 with cryoprotectants	Common carp (*Cyprinus carpio*)	Reduced microbial growth, total volatile basic nitrogen, and moisture for samples stored at superchilled conditions with cryoprotectants. Increased preservative impact of superchilling storage at −3 °C, especially when combined with cryoprotectants.	[100]
Clove essential oil enriched ice glazing	−1	Sea bass (*Dicentrarchus labrax*)	Considerable preservation of sensorial attributes during 24 days, when compared to control samples. Lower microbial and chemical degradation when superchilled. The preservation potential of the process increases with the concentration of essential oil.	[6]
Modified atmosphere (high CO_2_) (MAP)	−3	Swimming crab (*Portunus trituberculatus*)	Shelf life of crab was increased from 10–15 days, in conventionally superchilled samples, to 15–20 days in samples stored in superchilling under a modified atmosphere of 60–80% CO_2_. Lower bacterial growth and total volatile basic nitrogen in samples stored in MAP.	[95]
Modified atmosphere (high CO_2_ and N_2_) (MAP)	−1.7	Atlantic cod (*Gadus morhua*)	Shelf life: iced storage, 15 days; MAP iced storage, 21 days; air superchilling storage and MAP superchilling storage, >32 days. Total volatile basic nitrogen values remained below the EU limit after 34 days in superchilled samples. Lower aerobic viable counts but higher CFU/g of *Photobacterium* spp. in MAP samples.	[97]
Modified atmosphere (MAP) and chitosan treatment	−1	Atlantic cod (*Gadus morhua*)	Chitosan did not alter the sensory characteristics, freshness, or shelf life of the product. Decrease in total viable counts and total specific spoilage organism counts immediately after application of chitosan. Lower bacterial diversity in chitosan-treated samples. Lower total volatile basic nitrogen in MAP samples. Extension of 3–4 days of shelf life in MAP samples.	[86]
Modified atmosphere (high CO_2_) (MAP)	−1.3	Turbot (*Scophthalmus maximus*)	Superchilling storage with high CO_2_ (60–70% CO_2_) maintained better results in organoleptic, microbiological, and chemical parameters during storage.	[94]
Gelatin active coating with eugenol emulsion	−0.9	Chinese seabass (*Lateolabrax maculatus*)	Lower values of total volatile basic nitrogen, total viable count, H_2_S-producing bacteria, *Pseudomonas* spp., and psychrophilic bacteria in superchilled samples. The presence of eugenol in the coating showed improved efficiency in inhibiting product deterioration.	[87]
High-pressure processing (300 MPa)	−4	Mitten crab (*Eriocheir sinensis*)	High drip loss. Aerobic plate counts below the high-quality upper limit of 5 log CFU/g after 4 weeks. Total volatile basic nitrogen under 30 mg/100 g (maximum recommended) for 3 weeks. Extension of shelf life from 7 days (when refrigerated at 4 °C) to 3 weeks when superchilled and processed with high pressure.	[88]

**Table 3:** Compilation of studies on the impact of irradiation techniques on the quality and preservation of seafood.

Radiation Dose/Type	Food Matrix	Results	Reference
2, 4, 6, 8, and 10 kGy/EBI	Shrimp (*Solenocera melantho*)	Weight loss. Decrease in chewiness with increasing radiation. Reduced concentration of polyphenol oxidase. Strong bactericidal effect observed, increasing alongside radiation dose. Destruction of shrimp muscle above 6 kGy.	[122]
2, 4, 6, 8, and 10 kGy/EBI	Gazami crab (*Portunus trituberculatus*)	Changes in the composition of microbial communities. Decrease in bacterial variety. Proteobacteria dominated microflora above 4 kGy. *Psychrobacter* only inhibited above 8 kGy. The recommended dose to achieve bactericidal aims defined at 6 kGy.	[18]
1.5, 3, and 4.5 kGy/gamma	Nile tilapia (Oreochromis niloticus), herring *, mackerel *	Decrease in total viable bacteria. At 4.5 kGy, reduction in *Streptococcus*, *Staphylococcus*, yeasts, and molds below detectable values. Superior bactericidal activity at higher radiation doses. Considerably higher values of peroxide and TBA in irradiated samples. Reduced organoleptic score in samples irradiated with 4.5 kGy.	[126]
1, 3, 5, and 7 kGy/gamma	Silver carp (*Hypophthalmichthys molitrix*)	Reduced peroxide, TBA, and TVB-N values in irradiated samples. Up to 2 log CFU/g of reduction in irradiated samples after 15 days of storage. Increase in lipid oxidation and development of unpleasant odors. Reduced lightning index and superior yellowish color in treated samples. Increased softness, reduced chewiness, and hardness. Up to 3 days of shelf life-extension.	[127]
0.5, 1, 2, and 3 kGy/EBI	Atlantic salmon *	Reduced TVB-N values in irradiated samples. Increased TBA values are higher in treated samples. Inhibition of bacterial growth proportional to the radiation dose. Unpleasant color and odor at higher doses. No significant sensorial changes in doses below 2 kGy. Inhibition of bacterial growth.	[120]
1, 2, 4, and 6 kGy/gamma	Blue swimming crab (*Portunus pelagicus*)	Reduction in total viable counts. Elimination of *Vibrio cholerae* and *Vibrio vulnificus.* Inactivation of *Listeria monocytogenes*.	[121]

Legend: * no binomial classification of species provided by the study.

**Table 4:** Compilation of studies on the impact of high-pressure processing on the quality and preservation of seafood.

Pressure Applied	Food Matrix	Results	Reference
150, 300, and 450 MPa	Cod (*Gadus morhua*) and salmon (*Salmo salar*)	Efficient microbial reduction in samples treated with 450 MPa. Greater impact on color and cooked appearance when 300 and 450 MPa were used. Higher doses produced changes in all sensorial criteria. Increased lipid oxidation in salmon.	[133]
400, 500, and 600 MPa	Atlantic cod *	Reduction in total viable counts. Increased antibacterial activity at higher pressures. Extension of shelf life in all HPP-treated samples beyond 49 days of storage. HPP increased drip loss of product.	[15]
300 MPa	Sea bass (*Dicentrarchus labrax*)	pH increased after treatment. Sensorial alterations, increased lightness and hardness. HPP reduced overall acceptability. No increase in lipid oxidation was detected. Reduction in total viable bacteria, *Pseudomonas* spp., *Enterobacteriaceae*, and lactic acid bacteria. The shelf life increased from 5 to 9 days, based on the sensorial evaluation.	[136]
250 and 350 MPa	Hilsa (*Tenualosa ilisha*)	TBARS and TVB-N reduction in pressure-treated samples. Reduced lipid oxidation and TMA values. Lipid oxidation is higher at 350 MPa than 250 MPa. Modification and reduced acceptability of color characteristics of the product. Textural alterations. A 15-day increase in shelf life period.	[138]
200 and 500 MPa	Cod (*Gadus morhua*), salmon (*Salmo salar*), and mackerel (*Scomber scombrus*)	Significant bacterial inhibition in cod and mackerel. Mackerel shelf life extended from 8 to over 19 days. Cod shelf life extended from 15 to 21 and over 26 days for samples treated with 200 and 500 MPa, respectively. Increased lipid oxidation in all pressurized matrixes, especially those treated with 500 MPa.	[129]
100, 300, and 500 MPa	Mackerel (*Scomber* spp.)	Bacterial inhibition is proportional to the pressure applied. Decrease in total viable counts and H_2_S-producing bacteria. Negative impact on color. Increased hardiness in samples pressurized with 500 MPa. Changes in color and texture but no impact on lipid oxidation.	[128]

Legend: * no binomial classification of species provided by the study.

**Table 5:** Compilation of studies on the impact of biopreservative techniques on the quality and preservation of seafood.

Product	Biopreservative Agent	Results	Reference
Hake *	*Lacticaseibacillus paracasei* L26 and *Bifidobacterium lactis* B94	Lower total viable counts, H_2_S-producing bacteria, and total volatile basic nitrogen. TVB-N values below the limit of acceptability after 15 days. Over one week of extension of shelf life. Increase in probiotic cultures in the product.	[151]
Hake (*Merluccius hubbsi*)	*Enterococcus mundtii* STw38	Low values of total mesophilic counts (1.5 log cycles) compared to control (4.0 log cycles). Decrease in enterococci population for the initial 3 days, with recovery to inoculation levels afterward.	[149]
Ribbonfish (*Trichiurus lepturus*)	*Lactobacillus plantarum* SKD4 cell-free supernatant and *Pediococcus stilesii* SKD11 cell-free supernatant	Slight acidification of the product. Significant inhibition of bacterial growth. Low trimethylamine (TMA) values during storage. Diminished changes in color values. Conservation of sensorial characteristics throughout storage.	[154]
*Litopenaeus vannamei* (Shrimp)	*Lactobacillus plantarum* AB-1 and *Lactobacillus casei*	Higher sensory scores in co-cultured samples. Total volatile basic nitrogen under 30 mg/100 g limit for 8 days (5 days in control samples). Lower pH.	[155]
Horse mackerel *	*Lactobacillus sakei* ATCC 15521	Inhibition of bacterial growth, up to 1.5 log CFU/g. Typical bacteriostatic effect. Lower total volatile basic nitrogen and pH values.	[167]
Salmon *	Bacteriocin *Enterococcus faecalis L04*	Foodborne pathogen and food spoilage bacteria inhibition. Reduced total viable counts, lipid oxidation, and TVB-N values. Better maintenance of product quality during storage in refrigerated conditions. Preservation of sensorial characteristics.	[168]
Salmon *	*Carnobacterium maltaromaticum* SF1944, *Lactococcus piscium* EU2229, *Leuconostoc gelidum* EU2249, *Vagococcus fluvialis* CD264, *Carnobacterium inhibens* MIP2551, and *Aerococcus viridans* SF1044	Sensorial characteristics remained desirable for extended periods in samples treated with *V. fluvialis.* Strong, undesirable, acidification of samples inoculated with *L. piscium* or *L. gelidum*. Inhibited spoilage bacteria growth. Inhibition of *Listeria monocytogenes* growth.	[156]
Olive flounder (*Paralichthys olivaceus*)	Bacteriophage Spp001	Shelf life-extension from <4 to 14 days. Inhibition of bacterial growth, both total viable count and specific spoilage organisms. Preservation of good sensorial characteristics	[161]
Tuna *	Bacteriophage FSP1	No significant impact on total viable cell counts. Considerable inhibition of *Morganella morganni* cells. Reduced levels of histamine accumulation.	[162]
Atlantic horse mackerel (*Trachurus trachurus*)	Bacteriophage AZT6	Reduction in *Serratia* population by up to 90% during fish storage. Similar total viable counts to control.	[163]
Rainbow trout *(Salmo irideus)*	Bacteriophages Ah1, Pf1, Psp6, Ro1, Cf1, and Lm1	Inhibition of mesophilic aerobic bacteria growth. Samples treated with the cocktail remained under 10^5^ CFU/g for 3 days longer than control samples.	[165]

Legend: * no binomial classification of species provided by the study.

**Table 6:** Main advantages and disadvantages of the mentioned techniques.

Technique	Properties
Biodegradable films, edible coatings, and natural preservatives [10,45,46,47,48,49,50,51,52,53,54,55,56,57,58,59,60,61,62,63,64,65,66,67,68,69,70,71,72,73,74,75,76,77,78,79,80,81,82,83]	+Strong antibacterial activity +Safe +Biodegradable +All-natural final product +Great variety of candidate compounds +Easy to implement +Can add nutritional value and health claims to the product −Can result in strong organoleptic changes
Superchilling [6,13,84,85,86,87,88,89,90,91,92,93,94,95,96,97,98,99,100]	+Considerable increase in shelf life +Strong inhibition of bacterial growth +Preservation of most sensorial characteristics +Great potential if used in combination with other techniques such as MAP −Physical degradation if temperatures applied are non-optimal −Optimal temperature varies depending on matrix −Short optimal temperature interval
Ozonation [96,101,102,103,104,105,106,107,108,109,110,111,112,113,114,115,116]	+Versatile disinfectant +Activity against bacterial spores +Various forms of application +Sensorial preservation +Potential to reduce the presence of toxins such as diarrhetic shellfish toxins +Becoming progressively cheaper −Few studies on its application in seafood or other solid foods −Demands the acquisition of specialized equipment −Increases product manufacture cost
Irradiation [18,117,118,119,120,121,122,123,124,125,126,127]	+Low-intensity radiation preserves product characteristics +High-intensity radiation has strong antibacterial activity −High-intensity radiation increases TBARS values and results in changes in color, taste, texture, cohesiveness, and resilience −Impact on consumer health perceived as negative −Very expensive equipment and maintenance
High-pressure processing [14,15,128,129,130,131,132,133,134,135,136,137,138]	+Antibacterial activity increases with higher pressures +Potential to inactivate spores +Inactivation of allergens +Significant shelf life-extension −Sensorial impact at high pressures −Optimal pressure depends on product type −Very expensive equipment and maintenance
Hyperbaric storage [139,140,141,142,143]	+Low operating costs +Energetically efficient +Significant shelf life-extension +Preservation of sensorial characteristics +Maintenance of muscular structure and conservation of water holding and drip loss properties −Few studies −No commercial equipment available
Biopreservation [17,144,145,146,147,148,149,150,151,152,153,154,155,156,157,158,159,160,161,162,163,164,165,166,167,168]	+Lactic acid bacteria can add nutritional value to the product +Bacteriophages can be used to target specific bacteria +Beneficial bacteria is preserved −LAB activity might result in undesirable sensorial changes −Acidification of the product −Some doubts regarding the safety of bacteriophages exist

Legend: +, advantage of the technique; −, disadvantage of the technique.
